# Supplementary material for: Effects of using mobile augmented reality for simple interest computation in a financial mathematics course
Source: PeerJ Comput Sci. 2021 Jun 29;7:e618. doi: 10.7717/peerj-cs.618 (PMC8279137; doi:10.7717/peerj-cs.618)
Supplement: Supplemental Information 2 [file peerj-cs-07-618-s002.docx]

**The first survey (Pre-test)**

| **General Data** | | | | | |
| --- | --- | --- | --- | --- | --- |
| **Name (s)**: | **Surname:** |  | | | |
| **Age:** |  | | | | |
| **Gender:** | o (Male) | | o (Female) | | |
| **ARCS Professor** | | | | | |
| Please think about each statement concerning the professor session you have just participated and indicated how true it is. Give the answer that truly applies to you, and not what you would like to be true, or what you think others want to hear. Use the following values to indicate your response to each item: 1=N*ot true,* 2=*Slightly true,* 3=*Moderately true,* 4=*Mostly true,* and 5=*Very true.* | | | | | |
|  | **1** | **2** | **3** | **4** | **5** |
| **Attention (A)** |  |  |  |  |  |
| A1. The quality of the materials used helped to hold my attention. |  |  |  |  |  |
| A2. The way the information was organized helped keep my attention. |  |  |  |  |  |
| A3. The variety of lectures, exercises, and illustrations helped keep my attention on the explanations. |  |  |  |  |  |
| **Relevance (R)** |  |  |  |  |  |
| R1. It is clear to me how the content of this lesson is related to things I already know. |  |  |  |  |  |
| R2. The content and style of lesson explanations convey the impression that being able to work with is worth it. |  |  |  |  |  |
| R3. The content of the lesson will be useful to me. |  |  |  |  |  |
| **Confidence (C)** |  |  |  |  |  |
| C1. As I worked with this lesson, I was confident that I could learn how to compute simple interest well. |  |  |  |  |  |
| C2. After working with this lesson for a while, I was confident that I would be able to pass a test about simple interest. |  |  |  |  |  |
| C3. The excellent organization of the content helped me be confident that I would learn about simple interest. |  |  |  |  |  |
| **Satisfaction (S)** |  |  |  |  |  |
| S1. I enjoyed working with this lesson so much that I was stimulated to keep on working. |  |  |  |  |  |
| S2. I really enjoyed working with this simple interest lesson. |  |  |  |  |  |
| S3. It was a pleasure to work with such a well-designed simple interest explanation. |  |  |  |  |  |
