# Supplementary material for: Effects of using mobile augmented reality for simple interest computation in a financial mathematics course
Source: PeerJ Comput Sci. 2021 Jun 29;7:e618. doi: 10.7717/peerj-cs.618 (PMC8279137; doi:10.7717/peerj-cs.618)
Supplement: Supplemental Information 3 [file peerj-cs-07-618-s003.docx]

**Primer cuestionario (Pre-test)**

| **Datos Generales** | | | | | |
| --- | --- | --- | --- | --- | --- |
| **Nombre(s)**: | **Apellidos:** |  | | | |
| **Edad:** |  | | | | |
| **Sexo:** | o (Masculino) | | o (Femenino) | | |
| **ARCS para el Profesor** | | | | | |
| Por favor, piensa en cada una de las preguntas con respecto a la sesión en la que acabas de participar con el profesor, además, indica que tan cierta es. Ofrece la respuesta que verdaderamente aplique para ti, y no la que tu quisieras que fuera la correcta, o la que crees que otros quisieran escuchar. Utiliza los siguientes valores para indicar la respuesta a cada pregunta: 1=N*o es verdad,* 2=*Ligeramente verdadero*, 3=*Moderadamente verdadero,* 4=*Mayormente verdadero, y* 5=*Muy verdadero.* | | | | | |
|  | **1** | **2** | **3** | **4** | **5** |
| **Atención (A)** |  |  |  |  |  |
| A1. La calidad de los materiales utilizados ayudó para mantener mi atención. |  |  |  |  |  |
| A2. La forma en que la información fue organizada ayudó a mantener mi atención. |  |  |  |  |  |
| A3. La variedad de lecturas, ejercicios, e ilustraciones, me ayudó a mantener la atención en las explicaciones. |  |  |  |  |  |
| **Relevancia (R)** |  |  |  |  |  |
| R1. Es claro para mí que el contenido de esta lección se relaciona con temas que ya conocía. |  |  |  |  |  |
| R2. El contenido y el estilo de las explicaciones de la lección dan la impresión de que vale la pena trabajar con ellas. |  |  |  |  |  |
| R3. El contenido de la lección me será útil. |  |  |  |  |  |
| **Confianza (C)** |  |  |  |  |  |
| C1. Mientras trabajaba en la lección, confiaba en que podía aprender bien como calcular el interés simple. |  |  |  |  |  |
| C2. Después de trabajar con la lección por un rato, estaba confiado en que podía aprender bien como calcular el interés simple. |  |  |  |  |  |
| C3. La excelente organización de los contenidos me ayudó a tener confianza de que podía aprender sobre interés simple. |  |  |  |  |  |
| **Satisfacción (S)** |  |  |  |  |  |
| S1. Disfruté tanto trabajar con esta lección que me gustaría seguir trabajando en ella. |  |  |  |  |  |
| S2. Realmente disfruté trabajar con esta lección sobre interés simple. |  |  |  |  |  |
| S3. Fue un placer trabajar con las explicaciones tan bien diseñadas sobre interés simple. |  |  |  |  |  |
