# Supplementary material for: Effects of using mobile augmented reality for simple interest computation in a financial mathematics course
Source: PeerJ Comput Sci. 2021 Jun 29;7:e618. doi: 10.7717/peerj-cs.618 (PMC8279137; doi:10.7717/peerj-cs.618)
Supplement: Supplemental Information 4 [file peerj-cs-07-618-s004.docx]

**The second survey (Post-test)**

| **General Data** | | | | | |
| --- | --- | --- | --- | --- | --- |
| **Name (s)**: | **Surname:** |  | | | |
| **Age:** |  | | | | |
| **Gender:** | o (Male) | | o (Females) | | |
| **ARCS SICMAR** | | | | | |
| Please think about each statement concerning the SICMAR you have just used and indicated how true it is. Give the answer that truly applies to you, and not what you would like to be true, or what you think others want to hear. Use the following values to indicate your response to each item: 1=*Not true,* 2=*Slightly true,* 3=*Moderately true,* 4=*Mostly true,* and 5=*Very true.* | | | | | |
|  | **1** | **2** | **3** | **4** | **5** |
| **Attention (A)** |  |  |  |  |  |
| A1. The quality of the contents displayed helped to hold my attention. |  |  |  |  |  |
| A2. The way the information was organized (buttons, menus) helped keep my attention. |  |  |  |  |  |
| A3. The variety of 2D models and interactions helped keep my attention on the explanations. |  |  |  |  |  |
| **Relevance (R)** |  |  |  |  |  |
| R1. It is clear to me how the content of SICMAR is related to things I already know. |  |  |  |  |  |
| R2. The content and style of explanations used by SICMAR convey the impression that being able to work with is worth it. |  |  |  |  |  |
| R3. The content about simple interest will be useful to me. |  |  |  |  |  |
| **Confidence (C)** |  |  |  |  |  |
| C1. As I worked with SICMAR, I was confident that I could learn how to compute simple interest well. |  |  |  |  |  |
| C2. After working with SICMAR for a while, I was confident that I would be able to pass a test about simple interest. |  |  |  |  |  |
| C3. The excellent organization of SICMAR helped me be confident that I would learn about simple interest. |  |  |  |  |  |
| **Satisfaction (S)** |  |  |  |  |  |
| S1. I enjoyed working with SICMAR so much that I was stimulated to keep on working. |  |  |  |  |  |
| S2. I really enjoyed working with SICMAR. |  |  |  |  |  |
| S3. It was a pleasure to work with such a well-designed application. |  |  |  |  |  |
| **SICMAR TAM** | | | | | |
| Please select the number that best represents how do you feel about SICMAR acceptance: 1=*Strongly disagree*, 2=*Disagree*, 3=*Neutral*, 4=*Agree*, 5=*Strongly agree*. | | | | | |
|  | **1** | **2** | **3** | **4** | **5** |
| **Perceived Usefulness (PU)** |  |  |  |  |  |
| PU1. I could improve my learning performance by using SICMAR |  |  |  |  |  |
| PU2. I could enhance my simple interest proficiency by using SICMAR |  |  |  |  |  |
| PU3. I think SICMAR is useful for learning purposes. |  |  |  |  |  |
| PU4. By using SICMAR, it will be easy to remember the concepts related to the calculation of simple interest. |  |  |  |  |  |
| **Perceived Ease of Use (PEU)** |  |  |  |  |  |
| PEU1. I think SICMAR is attractive and ease of use |  |  |  |  |  |
| PEU2. Learning to use SICMAR was not a problem for me due to my familiarity with the use of technology. |  |  |  |  |  |
| PEU3. The markers detection was fast. |  |  |  |  |  |
| PEU4. The tasks related to controls manipulation were simple to execute. |  |  |  |  |  |
| PEU5. I was able to locate areas for conversions and calculations quickly. |  |  |  |  |  |
| **Intention to Use SICMAR (ITU)** |  |  |  |  |  |
| ITU1. I want to use the app in the future if I have the opportunity. |  |  |  |  |  |
| ITU2. The main concepts of SICMAR can be used to learn other topics. |  |  |  |  |  |
| **SICMAR Quality** | | | | | |
| Please select the number that best represents how do you feel about SICMAR quality: 1=*Not at all*, 2=*A little*, 3=M*oderate*ly, 4=*Much*, 5=*Very much*. | | | | | |
|  | **1** | **2** | **3** | **4** | **5** |
| **Quality questions** |  |  |  |  |  |
| Q1. SICMAR showed all the concepts explained by the teacher. |  |  |  |  |  |
| Q2. The results obtained with SICMAR were correct. |  |  |  |  |  |
| Q3. The colors used for conversions were adequate. |  |  |  |  |  |
| Q4. The texts and numbers displayed by SICMAR were legible. |  |  |  |  |  |
| Q5. The size of the buttons allowed SICMAR correct manipulation. |  |  |  |  |  |
| Q6. SICMAR velocity of response to carry out the calculations was fast. |  |  |  |  |  |
| Q7. The illumination of the place was adequate. |  |  |  |  |  |
| Q8. The manipulation of the electronic device I use was straightforward. |  |  |  |  |  |
| Q9. Markers’ manipulation was easy. |  |  |  |  |  |
| Q10. The manipulation of the device in conjunction with the markers was easy. |  |  |  |  |  |
