# Supplementary material for: Effects of using mobile augmented reality for simple interest computation in a financial mathematics course
Source: PeerJ Comput Sci. 2021 Jun 29;7:e618. doi: 10.7717/peerj-cs.618 (PMC8279137; doi:10.7717/peerj-cs.618)
Supplement: Supplemental Information 5 [file peerj-cs-07-618-s005.docx]

| **Pre-test** | **Post-test** |
| --- | --- |
| Isabel deposits $5,000 in a bank account that offers a simple interest of 6% per year. How much interest will Isabel receive per month of deposit?  **R.** $ 25.00 (using equation 1). | Calculate the simple interest on a loan of $8,500 to pay in 91 days, with a simple annual interest of 18%.  **R.** $386.75 (using equation 1). |
| John deposited $25,000 in a banking institution, and 18 months later, the balance in his account is $26030. What was the simple annual interest rate?  **R.** 2.7466% (using equation 4). | Alex applied for a loan of $3,000.00 and paid $3,400.00 after seven months. What was the simple annual interest rate?  **R.** 22.8571% (using equation 4). |
