# Supplementary material for: Effects of using mobile augmented reality for simple interest computation in a financial mathematics course
Source: PeerJ Comput Sci. 2021 Jun 29;7:e618. doi: 10.7717/peerj-cs.618 (PMC8279137; doi:10.7717/peerj-cs.618)
Supplement: Supplemental Information 6 [file peerj-cs-07-618-s006.docx]

**Segundo Cuestionario (Post-test)**

| **Datos Generales** | | | | | |
| --- | --- | --- | --- | --- | --- |
| **Nombre (s)**: | **Apellidos:** |  | | | |
| **Edad:** |  | | | | |
| **Sexo:** | o (Masculino) | | o (Femenino) | | |
| **ARCS para SICMAR** | | | | | |
| Por favor, piensa en cada una de las preguntas con respecto al prototipo SICMAR que acabas de utilizar, e indica que tan cierta es. Ofrece la respuesta que verdaderamente aplique para ti, y no la que tu quisieras que fuera la correcta, o la que crees que otros quisieran escuchar. Utiliza los siguientes valores para indicar la respuesta a cada pregunta: 1=*No es verdad,* 2=*Ligeramente verdadero*, 3=*Moderadamente verdadero,* 4=*Mayormente verdadero, y* 5=*Muy verdadero.* | | | | | |
|  | **1** | **2** | **3** | **4** | **5** |
| **Atención (A)** |  |  |  |  |  |
| A1. La calidad de los contenidos desplegados me ayudó para mantener mi atención. |  |  |  |  |  |
| A2. La forma en que la información fue organizada (botones, menús) me ayudó a mantener mi atención. |  |  |  |  |  |
| A3. La variedad de modelos 2D y las interacciones me ayudó a mantener la atención en las explicaciones. |  |  |  |  |  |
| **Relevancia (R)** |  |  |  |  |  |
| R1. Es claro para mí cómo el contenido de SICMAR se relaciona con temas que ya conocía. |  |  |  |  |  |
| R2. El contenido y el estilo de las explicaciones utilizadas en SICMAR dan la impresión de que vale la pena trabajar con ellas. |  |  |  |  |  |
| R3. El contenido sobre interés simple me será útil. |  |  |  |  |  |
| **Confianza (C)** |  |  |  |  |  |
| C1. Mientras trabajaba con SICMAR, confiaba en que podía aprender bien como calcular el interés simple. |  |  |  |  |  |
| C2. Después de trabajar con SICMAR por un rato, estaba confiado en que podía pasar un examen sobre como calcular el interés simple. |  |  |  |  |  |
| C3. La excelente organización de SICMAR me ayudó a tener confianza de que podía aprender sobre interés simple. |  |  |  |  |  |
| **Satisfacción (S)** |  |  |  |  |  |
| S1. Disfruté tanto trabajar con SICMAR que me gustaría seguir trabajando en ella. |  |  |  |  |  |
| S2. Realmente disfruté trabajar con SICMAR. |  |  |  |  |  |
| S3. Fue un placer trabajar con una aplicación tan bien diseñada. |  |  |  |  |  |
| **SICMAR TAM** | | | | | |
| Por favor seleccione el número que mejor representa cómo te sentiste acerca de la aceptación de SICMAR: 1=*Muy en desacuerdo*, 2=*No estoy de acuerdo*, 3=*Neutral*, 4=*De acuerdo*, 5=*Totalmente de acuerdo*. | | | | | |
|  | **1** | **2** | **3** | **4** | **5** |
| **Utilidad Percibida (PU)** |  |  |  |  |  |
| PU1. Podría mejorar mi rendimiento de aprendizaje usando SICMAR. |  |  |  |  |  |
| PU2. Podría mejorar mi competencia en interés simple usando SICMAR. |  |  |  |  |  |
| PU3. Creo que SICMAR es útil para el aprendizaje. |  |  |  |  |  |
| PU4. Al utilizar SICMAR, será fácil recordar los conceptos relacionados con el cálculo del interés simple. |  |  |  |  |  |
| **Facilidad de Uso Percibida (PEU)** |  |  |  |  |  |
| PEU1. Creo que SICMAR es atractivo y fácil de usar. |  |  |  |  |  |
| PEU2. Aprender a usar SICMAR no fue un problema para mí debido a mi familiaridad con el uso de la tecnología. |  |  |  |  |  |
| PEU3. La detección de marcadores fue rápida. |  |  |  |  |  |
| PEU4. Las tareas relacionadas con la manipulación de controles fueron sencillas de ejecutar. |  |  |  |  |  |
| PEU5. Pude ubicar las áreas para conversiones y cálculos rápidamente. |  |  |  |  |  |
| **Intención de Utilizar (ITU)** |  |  |  |  |  |
| ITU1. Quiero utilizar la aplicación en un futuro si se diera la oportunidad. |  |  |  |  |  |
| ITU2. Los principales conceptos de SICMAR pueden ser usados para aprender otros tópicos. |  |  |  |  |  |
| **Calidad de SICMAR** | | | | | |
| Por favor seleccione el número que mejor representa como te sentiste acerca de la calidad de SICMAR: 1=*De ningún modo*, 2=*Un poco*, 3=*Moderadamente*, 4=*Mucho*, 5=*Muchísimo*. | | | | | |
|  | **1** | **2** | **3** | **4** | **5** |
| **Preguntas sobre calidad** |  |  |  |  |  |
| Q1. SICMAR mostró todos los conceptos explicados por el profesor. |  |  |  |  |  |
| Q2. Los resultados obtenidos con SICMAR fueron correctos. |  |  |  |  |  |
| Q3. Los colores utilizados para las conversiones fueron adecuados. |  |  |  |  |  |
| Q4. Los textos y los números desplegados con SICMAR fueron legibles. |  |  |  |  |  |
| Q5. El tamaño de los botones permitió la correcta manipulación de SICMAR. |  |  |  |  |  |
| Q6. La velocidad de respuesta de SICMAR para realizar los cálculos fue rápida. |  |  |  |  |  |
| Q7. La iluminación del lugar fue adecuada. |  |  |  |  |  |
| Q8. La manipulación del dispositivo electrónico que utilice fue sencilla. |  |  |  |  |  |
| Q9. La manipulación de los marcadores fue fácil. |  |  |  |  |  |
| Q10. La manipulación del dispositivo en conjunto con los marcadores fue fácil. |  |  |  |  |  |
