# Supplementary material for: Effects of using mobile augmented reality for simple interest computation in a financial mathematics course
Source: PeerJ Comput Sci. 2021 Jun 29;7:e618. doi: 10.7717/peerj-cs.618 (PMC8279137; doi:10.7717/peerj-cs.618)
Supplement: Supplemental Information 7 [file peerj-cs-07-618-s007.docx]

**Code Book**

This document was created to explain how to read, understand, and convert the raw data presented in the file "*Data obtained from surveys*."

First, it is essential to mention that we used the Likert scale for all the surveys.

**General Information**

The first column was related to the age of the participants (students).

The second column included information about students' gender (5=Female and 1=Male). The first two columns share the information with both surveys. Remember, the surveys were conducted in a different session, so each time we collected the general data.

**First Survey**

Columns 3- 14 included the information collected with the **ARCS section** related to motivation in the professor's class. Columns 3-5 corresponded to attention items, columns 6-8 corresponded to relevance items, columns 9-11 corresponded to confidence items, and columns 12-14 corresponded to satisfaction items. Each value means the following 1=*Not true,* 2=*Slightly true,* 3=*Moderately true,* 4=*Mostly true,* and 5=*Very true.*

**Second survey**

Columns 15-26 included the information collected with the **ARCS section** related to motivation when students tested SICMAR. Columns 15-17 corresponded to attention items, columns 18-20 corresponded to relevance items, columns 21-23 corresponded to confidence items, and columns 24-26 corresponded to satisfaction items. Each value means the following 1=*Not true,* 2=*Slightly true,* 3=*Moderately true,* 4=*Mostly true,* and 5=*Very true.*

Columns 27-37 presented the information gathered with the **TAM section**. Columns 27-30 corresponded to Perceived Usefulness (PU) items, columns 31-35 corresponded to Perceived Ease of USE (PEU) items, and columns 36-37 corresponded to Intention to Use (ITU) SICMAR items. Each value means the following 1=*Strongly disagree*, 2=*Disagree*, 3=*Neutral*, 4=*Agree*, 5=*Strongly agree*.

Columns 38-47 presented the information obtained for each item in the Quality section. Each value means the following 1=*Not at all*, 2=*A little*, 3=M*oderate*ly, 4=*Much*, 5=*Very much*.
